# Carotta: Revealing Hidden Confounder Markers in Metabolic Breath Profiles

**DOI:** 10.3390/metabo5020344

**Published:** 2015-06-10

**Authors:** Anne-Christin Hauschild, Tobias Frisch, Jörg Ingo Baumbach, Jan Baumbach

**Affiliations:** 1 Computational Systems Biology Group, Max Planck Institute for Informatics, Saarbrücken 66123, Germany; E-Mail: tobias.frisch@fu-berlin.de; 2 Computational Biology Group, Department of Mathematics and Computer Science, University of Southern Denmark, Odense 5230, Denmark; E-Mail: jan.baumbach@imada.sdu.dk; 3 Department of Mathematics and Computer Science, Freie Universität Berlin, Berlin 14195, Germany; 4 Faculty of Applied Chemistry, Reutlingen University, Reutlingen 72762, Germany; E-Mail: joerg.baumbach@reutlingen-university.de

**Keywords:** breathomics, multicapillary column/ion mobility spectrometry, clustering, breath analysis

## Abstract

Computational breath analysis is a growing research area aiming at identifying volatile organic compounds (VOCs) in human breath to assist medical diagnostics of the next generation. While inexpensive and non-invasive bioanalytical technologies for metabolite detection in exhaled air and bacterial/fungal vapor exist and the first studies on the power of supervised machine learning methods for profiling of the resulting data were conducted, we lack methods to extract hidden data features emerging from confounding factors. Here, we present Carotta, a new cluster analysis framework dedicated to uncovering such hidden substructures by sophisticated unsupervised statistical learning methods. We study the power of transitivity clustering and hierarchical clustering to identify groups of VOCs with similar expression behavior over most patient breath samples and/or groups of patients with a similar VOC intensity pattern. This enables the discovery of dependencies between metabolites. On the one hand, this allows us to eliminate the effect of potential confounding factors hindering disease classification, such as smoking. On the other hand, we may also identify VOCs associated with disease subtypes or concomitant diseases. Carotta is an open source software with an intuitive graphical user interface promoting data handling, analysis and visualization. The back-end is designed to be modular, allowing for easy extensions with plugins in the future, such as new clustering methods and statistics. It does not require much prior knowledge or technical skills to operate. We demonstrate its power and applicability by means of one artificial dataset. We also apply Carotta exemplarily to a real-world example dataset on chronic obstructive pulmonary disease (COPD). While the artificial data are utilized as a proof of concept, we will demonstrate how Carotta finds candidate markers in our real dataset associated with confounders rather than the primary disease (COPD) and bronchial carcinoma (BC). Carotta is publicly available at http://carotta.compbio.sdu.dk [1].

## 1. Introduction

In the last decade, the field of breathomics, defined as the metabolomics study of human exhaled air, grew tremendously. One of the major goals is to non-invasively “sniff” biomarker molecules that are predictive for the biomedical fate of individual patients. These so-called personalized medicine (or precision medicine) approaches promise great hope to move the therapeutic windows to earlier stages of disease progression.

Analytical technologies that overcome the obstacles of exhaled air analysis, like humidity and variability, exist. The computational methods, especially for advanced statistical breathomics analysis, however, are still in their infancy. To pave the way for this technology towards daily usage in medical practice, these challenges remain to be addressed.

### 1.1. Analytical Technologies for Breathomics

Various high-throughput and high-resolution technologies have been developed over the last few years, producing tremendous amounts of increasingly complex data [2]. The major spectrometric techniques currently employed are gas chromatography-mass spectrometry (GC/MS) [3–5], electronic noses [6,7], proton transfer reaction-mass spectrometry (PTR-MS) [8,9] and ion mobility spectrometry (IMS) [10–14].

All such approaches are non-invasive and provide the potential for early and fast diagnosis, therapy monitoring and therapy optimization through identifying medically-relevant patterns in the spectrum of exhaled substances that are associated with certain (stages of) disease (progression). However, the sampling procedure remains a critical point for the majority of the methods [15]. Therefore, the on-site analysis of samples is a significant advantage of portable devices, like ion mobility spectrometry coupled to multi-capillary columns (MCC) or electric noses. Note that MCC/IMS devices potentially offer identifying the key components in the gathered samples, in contrast to electronic noses. The IMS technology was developed in the early 1970s and originally used for military applications [16,17] and the detection of drugs or explosives, e.g., at airports. The powerful combination with multi-capillary columns allows for many possible application opportunities, in particular in medicine [18–20] and biomedicine [21]. The main analytical advantages of the MCC/IMS technique are the ability to handle the moisture in exhaled air and the high sensitivity (detection limit at nanograms to picograms per liter) compared to other spectrometric techniques (e.g., GC/MS). Particularly, the short sampling time (about 10 s) and sample processing (about 5–10 min), as well as the robust and easy handling in every day practice make the MCC/IMS technique suited specifically for large-scale screening studies [19].

### 1.2. Motivation

The number and size of the datasets emerging from those studies evoke new challenges in terms of data management and analysis. Breathomics faces the traditional biomarker research barrier, just as many other omics technologies: a lack of robust statistical data analysis methods hinders translation to the world outside laboratories. Tools for visualization [19], preprocessing and peak detection [22,23] have been developed and various explorative statistical inference measures (e.g., Mann–Whitney U-Test or correlation) [12] and dimension reduction (principal component analysis, PCA) have been applied. The usage of more sophisticated learning methods and robust evaluation remains the minority, however [11,24–26]. Furthermore, most computational breathomics studies focus on the separation of a set of subjects into previously known subgroups. However, as with related omics technologies, the metabolic patterns of the human exhaled air are influenced by various sources of disturbance originating from the environment or nutrition, for instance. These known or unknown confounding factors might form hidden structures in the data that conceal the important information. They may, however, be useful when they relate to disease subtypes, varying phenotypes or concomitant diseases (secondary disorders) emerging within the group of volunteers.

In the last few years, the field of breathomics has opened up to the advantages of modern statistical learning approaches [25,26]. Some very recent studies (mostly in 2014) utilized unsupervised learning methods to analyze breath gas in order to define adult asthma endotypes [27], compare human body chemistry between breath and skin [28] and to identify pulmonary diseases sub-phenotypes [29]. This emphasizes the emerging need for such technology. However, none of the existing studies emerged with a software or bioinformatics toolbox addressing the community’s need for automatic unsupervised processing of breathomics data. In addition, in breath analysis, multi-dimensional clustering that allows for identifying groups of metabolites associated with groups of patients was not applied yet.

Existing work further lacks in-depth evaluations using, for instance, the F-measure together with disease annotation data (gold standards). Parameters were usually set rather arbitrarily instead of systematically by utilizing internal separation measures, such as the silhouette value. The quickly emerging breathomics field requires such solutions to efficiently screen large-scale data for hidden metabolite profiles associated with sub-groups of patients, as they are potential markers for confounders or secondary diseases.

A large body of bioinformatics approaches exists, but has not been designed for breathomics data. Consequently, they were not employed in the breathomics community and have not been evaluated sufficiently yet. One of the main reasons for the tentative usage of modern learning methods is the fact that most of the various software packages for more advanced analysis require expert knowledge in the area of statistics and often even expertise in programming. Popular examples are graphical tools, like Weka [30] and RapidMiner [31], or statistical learning environments, like R [32]. Other promising approaches for multi-dimensional clustering exist, such as bi-clustering or co-clustering (see, e.g., [33,34]), but they do not yet provide graphical user interfaces to visually explore the results systematically in response to changing input parameter sets. Therefore, a comprehensive and user-friendly software is needed to fill the gap between the quickly emerging breathomics datasets and the requirements of current breath data analysis.

This encouraged us to design Carotta, a software application that provides easy access to advanced unsupervised learning analysis specifically designed for breath data analysis. We are addressing two main goals, a user-friendly front-end, including several visualization options, as well as a flexible and modular back-end that is open for functional extensions. Carotta guides the user through the different steps of unsupervised learning analysis, starting with the similarity function, clustering, cluster quality evaluation, filtering and visualization by dimension reduction. Thereby, it offers biomedical researchers access to these techniques without requiring deeper knowledge of advanced learning techniques. The software application provides an intuitive way to process and analyze the data efficiently, reaching back to well established machine learning technologies in the background. The flexible plug-in system allows future methods to be added in a straight-forward fashion. Each step comes with an interactive visualization allowing for in-depth investigation of intermediate results directly in the user interface without the necessity to install and configure external software packages or libraries.

## 2. System and Implementation

**Figure 1:**
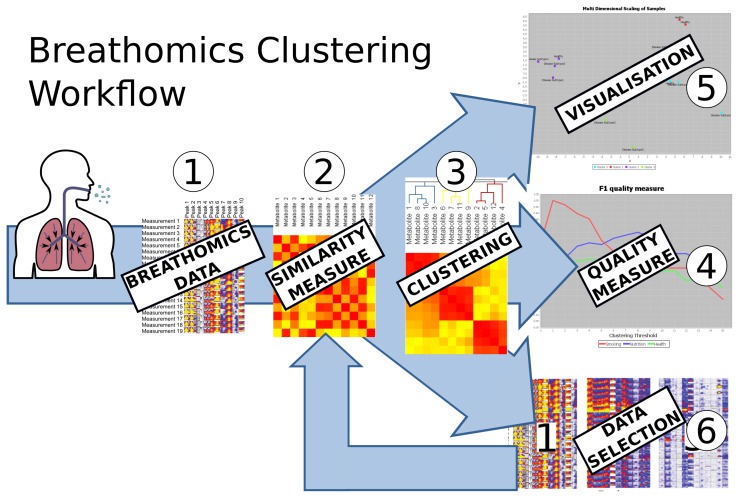
The carotta pipeline consists of several steps: (1) import of pre-processed data (see Section 2); (2) similarity calculation; (3) clustering; (4) clustering quality; (5) similarity or clustering visualization; (6) subset selection. Intermediate results of Steps 2–4 can be inspected, optimized and repeated at an arbitrary depth.

The carotta software framework provides interactive access pipelines revealing hidden structures from any kind of metabolomics data; see the general Carotta workflow in Figure 1.

In the first step of the carotta workflow, the data are imported into the system and displayed. Beforehand, the raw data have to be preprocessed by technology-specific pre-processing methods, such as baseline correction, de-noising, as well as peak detection (for MCC/IMS and GC/MS), such that a data matrix, as shown in Figure 1, is generated. In the future, we plan to integrated such pre-processing steps directly into Carotta as plugins. A review paper for such methods may be found in Smolinska *et al.* 2014 [25]. In Step 2, the pairwise relations of objects, either of study subjects (e.g., patients) or metabolites, can be calculated based on one of the incorporated measures (Pearson correlation coefficient, Spearman correlation coefficient or Euclidean distance [35]). See Section 5.1 for details.

**Figure 2:**
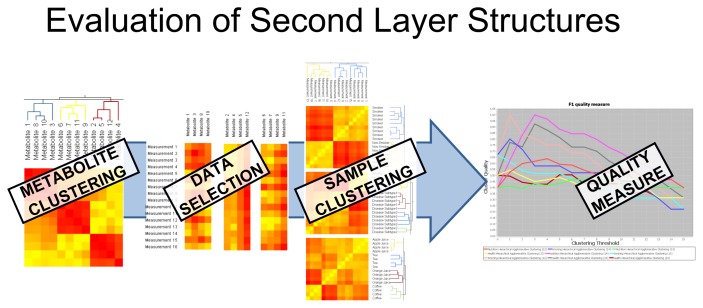
The subset selection allows for the analysis of the hidden structures in the data. Steps 2–4 from Figure 1 are repeated on a selected subset (or all subsets). Here, we separate artificial data with respect to the metabolite clusters discovered in the first layer. The second layer clustering of the samples (patients) now evaluates the association of each metabolite cluster to selected patient annotations (*i.e*., labels; here: “health”, “nutrition” and “smoking”). Finally, the F-measure plots show to what extent the metabolite clusters “explain” the different labels.

These pairwise relations are stored in a matrix and depicted by a heat map. All further steps require this matrix to present either a similarity or a dissimilarity; therefore, the dissimilarity matrix is converted into a similarity matrix, and *vice versa*, according to the needs of the following step. This is done as follows: the converted dissimilarity is defined as *d*(*x, y*) = *max*(|*P*|) – |*p*(*x, y*)|, where *P* is the matrix containing the original similarity and *p*(*x, y*) corresponds to the similarity of object *x* and *y*. The converted similarity is defined accordingly: *p*(*x, y*) = *max*(|*D*|) – |*d*(*x, y*)|. Further, these representations can be visualized in a two-dimensional scatter plot by using multi-dimensional scaling (MDS) [36]; see Figure 1, Step 5. In the next step, a clustering algorithm can be applied based on these pairwise relations. Two state-of-the-art clustering algorithms are integrated into the system, namely hierarchical agglomerative clustering (HAC) [37], which is based on pairwise dissimilarities, and transitivity clustering (TC) [38], which is based on similarities; details on the methodology are given in Section 5.2. Depending on the method of parameters (set of thresholds), the result of one clustering algorithm is a list of groupings, each corresponding to a certain threshold. We will refer to the set of all groupings as the clustering result and to each grouping as clustering. In Step 4, the value of these clusterings can be evaluated and compared by means of two quality measures, the silhouette value and the F-measure. One may now select one clustering result (for one threshold, which yielded optimal results, for instance) and visualize it using the MDS coordinates of the underlying similarity, as well as by means of a scatter plot color-coded by cluster. Finally, filtering methods can be utilized to select a subset of the data, for example a representative for each cluster or all objects of one cluster in a certain clustering. By repeating steps one to four on the selected subsets of the data, Carotta explores various layers of potentially hidden sub-structures. Especially the cross-clustering of samples and metabolites can reveal novel information; see Figure 2.

In short, Carotta can be used to split the set of metabolites into subsets (clusters), which, in turn, can be used individually to inspect their association with the primary outcome variable, *i.e*., the disease. This allows for eliminating large sets of metabolites, which correlate with potential confounding factors rather than the investigated disease (elimination of unimportant features). Most notably, Carotta automatizes these steps and provides intuitive means for intermediate result visualization.

### 2.1. Visualization

**Figure 3:**
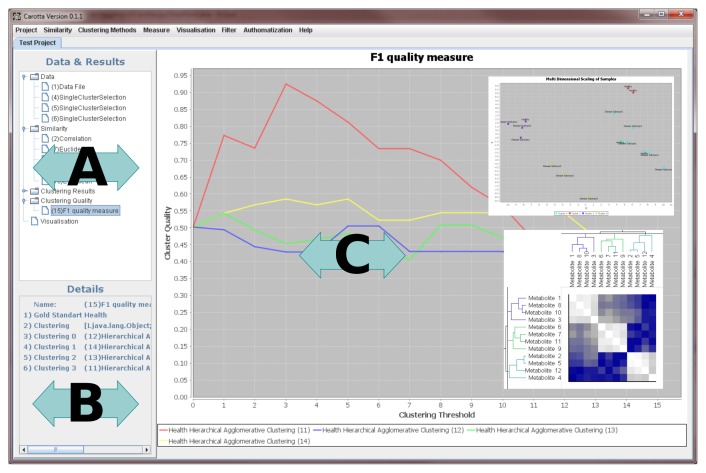
The graphical user interface is split into three basic regions: (**A**) the data and results area lists available (intermediate and final) results; (**B**) a “details” panel; (**C**) the main result visualization panel.

The graphical user interface (see Figure 3) is split into three basic regions: (1) the data and results area, showing a list of all generated results ordered in a tree-like structure; the categories correspond to the previously described processing steps (data, similarity, clustering results, clustering quality, visualization); (2) a “details” panel, reporting the parameter of the currently presented result; this also includes, for instance, general information on the dataset (such as the minimum and maximum values; (3) the main result visualization panel displays the results of the different intermediate steps, as well as the final results.

In the following, we will describe the visualization of each of the previously described steps in the graphical user interface in detail.

#### Data and similarity matrix

Each data or similarity matrix is displayed as a heat map tagged by the corresponding metabolite names and sample label, on the columns and rows, respectively. Labels can be changed to arbitrary annotation details included in the original data matrix.

#### Clustering

The heat map of the underlying similarity matrix is displayed in the center of the clustering result visualization. The rows and columns are sorted by the corresponding clustering. For hierarchical clustering, results can be inspected interactively by selecting a clustering threshold by sliding with the mouse in the two dendrograms. Leaf nodes correspond to clustered objects; inner nodes depict how the dataset is split (top down) or merged (bottom up) during the clustering. For transitivity clustering, one may manually adjust the threshold through a bar on the right side. Depending on the selected cut, but independent of the utilized clustering method, colors encode the resulting clusters. The axis labels are user definable.

#### Cluster quality

The quality of one or more clusterings can be evaluated for varying cuts/thresholds by using line plots. In the case of the external F-measure, the visualization depicts the comparison of the clustering to one or more user-selected class label(s). To identify a reasonable cut/threshold, the internal silhouette value measure may be applied (varying thresholds, but without the gold standard).

#### Multi dimensional scaling

The visualization of the similarity of a set of objects is provided by a customizable scatter plot, based on coordinates determined by the MDS. Besides the custom-defined labeling, the depiction of a clustering result can be colored according to a chosen threshold. This representation can give the first indication of whether a clustering is “good”.

### 2.2. Modularity and Extendibility

Carotta is open source. Due to its modular structure, new functionality can be integrated easily. Each of the previously described processing steps (similarity, clustering, cluster quality and visualization) can be expanded by additional methods. Java reflections guarantee a comfortable plug-in system that does not require any further editing of the previous code.

### 2.3. Import and Export

Convenient functions to export all intermediate and final results are included. The system provides the export of all visualizations described before. The user has the possibility to choose between different resolutions of the resulting portable network graphics (PNG) image file. Carotta further supports exporting into an MS Excel file (e.g., a similarity matrix or the results of the quality measure).

### 2.4. Language and Packages

The Carotta software package and associated software libraries are purely Java-based. The source code is available at the project website and underlies the Apache License Version 2.0. More information on the technical aspects can be found in the Supplementary Material and the following address: http://carotta.compbio.sdu.dk/ [1]. The following software packages have been used.

The TransClust package for transitivity clustering [39].
The HAC package for hierarchical agglomerative clustering [40].
The JExcelApi 2.6.12 parsing the excel sheet into the internal data structure [41].
The JFreeChart 1.0.14 visualization (clustering quality, scatter plot of MDS) [42].
The JHeatChart 0.6 creating the heat map [43].
The MDSJ calculation of the multi-dimensional scaling [44].
The Guava & Reflections & Javassist Google Core Libraries [45] and the Javassist [46] are used for the reflections technology.
The log4j 2.0 for logging and debugging [47].

## 3. Results and Discussion

To demonstrate the abilities of the Carotta clustering framework, we analyze two datasets, one artificial and one real world.

### 3.1. Artificial Data

The artificial dataset is dedicated to demonstrating and clarifying the capabilities of Carotta. It consists of 16 samples and 12 metabolites associated with three metabolite groups. Each of these groups is related to one of the three predefined labels of the samples: (1) “health” (values: healthy (five), disease Subtype 1 (five), disease Subtype 2 (six)); (2) “smoking” (values: smoker (nine), non-smoker (seven)); (3) “nutrition” (values: apple juice (four), tea (four), orange juice (four), coffee (four)). The label “health” is our primary outcome variable, while “smoking” and “nutrition” shall be considered as potential confounding factors. Supplementary Tables 1–3 show the corresponding mean and standard deviations used to generate normal distributions from which the artificial dataset was sampled. Note that these simulated data are highly idealized. This serves the sole purpose of exemplifying and clarifying Carotta’s use and functionality.

We will use this dataset to exemplify the power of Carotta. We first used the Pearson correlation coefficient to calculate the similarities between all metabolite occurrences, and clustered them by both HAC and TransClust. Independent of the clustering method, the silhouette value indicates, as expected, an optimum of three different metabolite clusters. As demonstrated in Figure 2, the full dataset is now split into three subsets, one for each cluster of correlating metabolites. Subsequently, for each cluster, as well as the full dataset, the Euclidean distance between all pairs of patient samples is computed (separately for each cluster). The three resulting clusterings gained from the metabolite clusters are compared against the clustering achieved with the full metabolite dataset using the F-Measure (see Section 5.3 for details).

**Figure 4:**
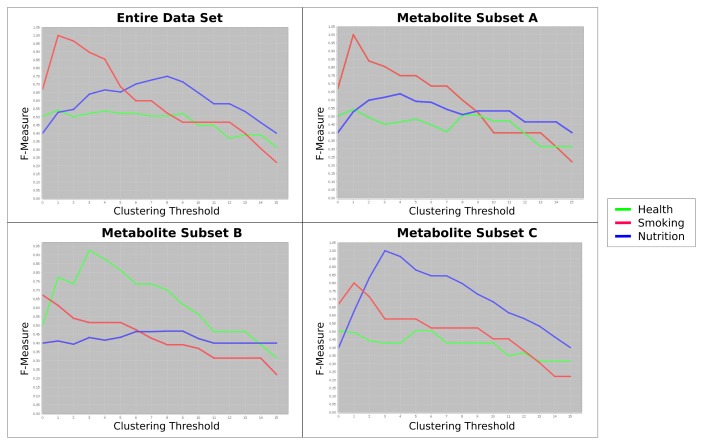
Carotta’s final output. The top left plots show the F-measure for different clustering thresholds on the full dataset, containing all metabolites. We observe a dominant effect of the confounder “smoking”, which overlays the main outcome variable “health”. The other three plots show the F-measure behavior over different thresholds for each cluster of correlated metabolites separately. They clearly reflect and dissect the labels “health”, “smoking” and “nutrition” now.

Figure 4 shows this evaluation of the clusterings in relation to the initially-designed class labels (“health”, “smoking” and “nutrition”). In this artificial example, the entire dataset is heavily confounded by the influence of the “smoking”-related metabolites. If we cluster the dataset using all metabolites, the effect of those metabolites related to “smoking” are too dominant (red curve). Consequently, we cannot detect the samples with the label “health” (green curve).

When we analyze the F-measure curves for the three clusters of metabolites separately, however, we may (1) detect this confounding effect and (2) reduce it. The metabolite Subset Clusterings A and C show perfect F-measures for “smoking” and “nutrition”, respectively. The metabolites clustered together in Subset B contain information to group the samples according to the label “health”.

### 3.2. COPD Data

COPD is an inflammatory lung disease characterized by a permanent blockage of airflow from the lungs, which is not fully reversible. The airways and lungs react to noxious particles or gases, like smoke from cigarettes or fuel, with an enhanced inflammatory response [48]. The World Health Organization (WHO) reported it as one of the most frequent causes of death. In the period between 2000 and 2011, the disease caused 5.8% of all deaths worldwide [49]. Even though it is a leading cause of morbidity and mortality worldwide, it is still widely under-diagnosed. Young *et al.* reported in 2009 that COPD is both a common and important independent risk factor for lung cancer [50]. Lung cancer is defined as an “uncontrolled cell growth in lung tissue, usually in the cells lining air passages” [51]. Two main subtypes are small cell lung cancer and non-small cell lung cancer [51]. They are diagnosed based on the microscopic visual appearance of the cells. The survival rate of patients within five years is less than 20% depending on the state of the carcinoma. Today, the majority of bronchial carcinoma is detected randomly during routine examinations.

Here, we study the exhalome of COPD patients using a dataset from [52]. It consists of metabolic maps from 42 COPD patients, 52 patients suffering from both, COPD and bronchial carcinoma, as well as 35 healthy controls. The patients’ breath was captured and analyzed using an ion mobility spectrometer coupled with a multi-capillary column, as introduced before. We identified 120 volatile organic compounds present in at least three of the patients’ measurements.

**Figure 5:**
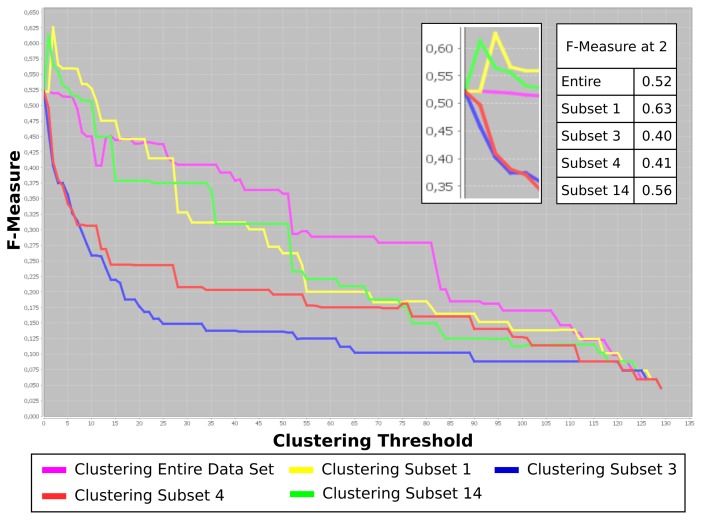
Comparison of the clustering results on the entire COPD dataset, *i.e*., using all metabolites, as well as the four most interesting metabolite clusters (two best and two of the worst). The plot shows the F-measure for different clustering thresholds computed against the disease annotation (COPD, COPD with bronchial carcinoma (BC) and healthy). The Y-axis corresponds to the clustering threshold, in this case the number of splits. Given three groups of patients in the annotation, we are particularly interested in the performance at clustering results at *T* ~ 2 (x-axis). This is shown in more detail in the zoomed cutout, as well as the table of F-measure values at this position. Two subsets of metabolites overlap with the patients’ disease annotation better than the clusterings based on the entire metabolite set. The two other metabolite subsets result in reduced F-measures, indicating a relation to confounding factors, in this case menthol (see the text).

This dataset was evaluated utilizing Carotta following the previously introduced workflow. At first, all 120 metabolites were clustered by HAC and the Pearson correlation (converted to dissimilarity, as explained above). Several thresholds (thus, varying numbers and sizes of clusters) were investigated, leading to an optimal result of *T* = 40. Subsequently, the set of metabolites was split into 40 subsets, one for each cluster of correlating metabolites. We now exclude all clusters with less than three compounds, leaving us with a total of 14 metabolite sets. Finally, the hierarchical agglomerative clustering was performed on the correlation matrix (converted to the distance matrix, as previously described) of the patients for each of these metabolite sets. Carotta subsequently evaluates the overlap of the patient clusters with the three patient groups over varying clustering thresholds using the F-measure. Figure 5 plots the results for four of the 14 metabolite subsets, as well as the results when using the entire set of metabolites. For better visualization, we restricted the figure to the five most interesting results: the entire metabolite set and the two best (highest F-measures), as well as two of the worst (lowest F-measures) performing metabolite sets.

We are given annotations for three groups of patients. Thus, we investigate the overlap of the clustering results at *T* ~ 2, corresponding to two splits of the data. One can see two metabolite clustering subsets, namely Subsets 1 and 14, that peak around three clusters. Both exceed the F-measure achieved using the full metabolite set.

The compounds within these clusters have been manually compared (via their specific peak coordinates) to the results of previous COPD studies utilizing supervised learning methodology [11]. Three of the compounds in Subset 1 were previously reported as potential biomarkers.

This shows that the presented stepwise multi-dimensional clustering approach points out putative COPD marker metabolites by using a purely unsupervised approach. In contrast, the metabolites in Subsets 3 and 4 show a rapid decrease in the F-measure for a growing number of clusters. The evaluation of the list of compounds within these clusters uncovered that these subsets contain the menthol trimer (Subset 3), as well as the menthol monomer and dimer (Subset 4) compounds, respectively. The occurrence of menthol in human exhaled air can be the result of various environmental and nutritional influences, for example tooth paste or candy.

This exemplifies where Carotta is useful: when we expect yet uncharacterized confounders to exist, which have an effect on the metabolic patterns, we like to detect and exclude them. The human exhaled air in particular can be influenced by various external factors, like nutrition and compounds in the environmental air. They do not need to be known *a priori*, however. Our menthol example in human breath from above serves as a proof of concept here.

Further analyses of the clustering results would be beneficial in the future. In particular, we need to investigate to what extent the elimination of putative confounding metabolites would improve the classification performance in a systematic statistical learning study. This clearly goes beyond the focus of this paper. We will address such aspects in future work.

## 4. Conclusions

We presented Carotta, a software for *de novo* detection of confounding factors and disease sub-types. It is open source and comes with an intuitive graphical user interface for unsupervised breathomics data analysis and visualization. The flexible back-end design supports easy extensions with plugins in the future, new clustering methods and statistics. It intuitively guides the user through four steps: (1) similarity matrix computation; (2) clustering; (3) clustering evaluation; and (4) results visualization and interpretation. This process does not require much prior knowledge or technical skills to operate and is therefore suitable for non-technical trained personnel. By means of an artificial dataset, we demonstrated the power and applicability of the Carotta software framework for revealing hidden structures and confounding factors (in a highly idealized setting). In addition, we exemplarily utilized Carotta to re-analyze a real-world example dataset on COPD. We demonstrated how Carotta helps with finding potential informative metabolite clusters containing substances also supported by previous studies. Most notably, it identified confounder metabolites (e.g., menthol), which are related to nutrition and the environment rather than to the primary outcome variable (disease annotation, *i.e*., COPD and lung cancer). The Carotta software framework offers easy access to extensive clustering analysis to non-technical personal working in the area of breathomics. It is publicly available at http://carotta.compbio.sdu.dk [1].

## 5. Methods

### 5.1. Dissimilarity and Similarity Measures

The pairwise relation of two data points is defined by a similarity or dissimilarity function. This function is how this relation is calculated within a high-dimensional space. Depending on the clustering approach, either the similarity or dissimilarity matrix is needed. Therefore, the analyzed similarity and dissimilarity matrices need to be converted accordingly. A similarity matrix is converted into a dissimilarity matrix as follows: The entries of the new matrix are defined as *d*(*x, y*) = *max*(|*P*|) – |*p*(*x, y*)|, where *P* is the matrix containing the original similarity and *p*(*x, y*) corresponds to the similarity of objects *x* and *y*. The similarity based on the dissimilarity is defined accordingly: *p*(*x, y*) = *max*(|*D*|) – |*d*(*x, y*)|.

#### Pearson Correlation

The Pearson correlation coefficient [35] is a measure of linear correlation. It is varying between −1 and 1, where −1 is negative correlation, 0 is no correlation and 1 is positive correlation.

(1) $$p(x,y)=\frac{\sum_{i=1}^{n}\left(X_{i}-\bar{X}\right)\;\left(Y_{i}-\bar{Y}\right)}{\sqrt{\sum_{i=1}^{n}{\left(X_{i}-\bar{X}\right)}^{2}}\sqrt{\sum_{i=1}^{n}{\left(Y_{i}-\bar{Y}\right)}^{2}}}$$

In the following, we focus on the absolute value of the correlation.

#### Spearman Correlation

A non-parametric version of the Pearson product-moment correlation is the Spearman correlation. The corresponding value estimates how well one variable can be described as a monotonic function of another variable. It varies between −1 and 1, where −1 is negative correlation, 0 is no correlation and 1 is positive correlation. It is defined as the Pearson correlation coefficient between the ranks of variables [53].

#### Euclidean Distance

The Euclidean distance [35] is the most commonly-used dissimilarity measure. It is defined by the following equation:

(2) $$d(x,y)=\sqrt{\sum_{i=1}^{n}{\left(x_{i}-y_{i}\right)}^{2}}$$

The function is given by the Pythagorean theorem and is always greater than zero, besides the two points being equal.

### 5.2. Unsupervised Statistical Learning

Unsupervised methods try to find hidden structures without incorporating external knowledge. Essentially, they identify groups (clusters) of data objects that are more similar to each other than to objects from other groups [37]. In the following section, we focus on two common clustering algorithms, namely hierarchical agglomerative clustering and transitivity clustering. We briefly introduce them in the following.

#### Hierarchical Agglomerative Clustering

The hierarchical agglomerative clustering (HAC) is one of the most widely-used clustering algorithms based on the dissimilarity of objects [37]. In contrast to the divisive “top down” approach, the first level of the HAC algorithm assigns every object to its own cluster. In an iterative process, the most similar (smallest distance) clusters are merged. This builds a hierarchy of similar elements resulting in a different set of clusters (clustering) for each step. The dissimilarity between two clusters of a set of objects of different coordinates are defined by certain agglomeration or linkage methods. Popular examples are the average- or complete-linkage specified as the average or the maximum of all pairwise dissimilarities of all objects between the two clusters, respectively. Please find the complete list of agglomeration methods in Supplementary Material Section 2. Each HAC run results in a set of *N* clusterings, where *N* is the number of objects to be grouped.

#### Transitivity Clustering

Transitivity clustering is based on the weighted transitive graph projection problem [38]. A given similarity matrix is interpreted as a weighted similarity graph and split into a cost graph by removing edges with weights below a user-given threshold. Such a putatively intransitive cost graph *G* = (*E, V* ) will be transformed into a transitive graph *G*′ by adding and removing a minimal number of edges. In practice, the edge weights are taken into account, yielding a cost function for edge modifications that is to be minimized. In 2010, Wittkokp *et al.* published an algorithm that tackles this NP-hard problem by combining exact and heuristic algorithms [39]. The threshold influences the number of clusters, as the average similarity of objects within one cluster is (provably) above the threshold, while the average similarity of the object from different clusters is below the threshold. Consequently, a high threshold leads to many small clusters, while a low threshold has few, but bigger clusters. The Transitivity Clustering software also provides a hierarchical clustering mode.

#### Application and Thresholds

Besides methodological delineation the main difference between the two approaches is the real-world interpretation of the threshold. In hierarchical clustering, it corresponds to the number of clusters. In contrast, in transitivity clustering, it corresponds to the similarity value *S*, for which the average similarity of all objects from different clusters is smaller than *S* (and the similarities between objects from the same cluster is higher than S, on average). The selection of the clustering method depends on the purpose of the study and the datasets at hand. Using hierarchical clustering usually appears beneficial if we may assume (or guess) a certain number of clusters. In datasets with few or no outliers, this might become problematic. If prior knowledge on a preferable similarity cutoff is available, transitivity clustering will be more appropriate. It is more robust to outliers, as it is independent of the number of clusters (*i.e*., outliers would end up as singletons).

### 5.3. Quality Measures

A clustering quality measure gives evidence of how well the groups of objects are separated by the clustering. Internal quality measures are based on the pairwise relation of the objects. In contrast, external indices compare the clustering result to a user-given gold standard, *i.e*., the primary outcome variable (in our case).

#### Silhouette Value

A prominent example for an internal quality measure is the silhouette value [54]. It evaluates how well an object fits into the associated cluster depending on the paired dissimilarity to the objects within its cluster in contrast to the objects in all other clusters. It is defined as follows:

(3) $$S(i)=\frac{b_{i}-a_{i}}{\text{max}\{a_{i},b_{i}\}}$$

Here, *a_i_* is defined as the average dissimilarity to all objects in the same cluster, while *b_i_* is the dissimilarity to the so-called neighbor cluster, which is the cluster of the next lowest average dissimilarity to *i*. The average of all object silhouette values is called the overall silhouette value of a clustering. The value varies between one and minus one. If all elements are well clustered, the result will be one.

#### F-measure

Let *K* be the gold standard defining a known grouping of the objects. The F-measure compares the clustering *C* to the gold standard, whereas *t_i,j_* denotes the number of common elements of *K_i_* and *C_j_*. The final F-measure among all clusters is varying between 0 and 1. While 0 corresponds to a poor overlap with the gold standard, 1 indicates a perfect match [55]. It is defined as follows:

(4) $$F-measure\;\left(C,K\right)=\frac{1}{\sum_{i=1}^{m}|K_{i}|}\sum_{i=1}^{m}\left(|K_{i}|*\underset{i\leq j\leq n}{\text{max}}\frac{2*t_{i,j}}{|C_{j}|+|K_{i}|}\right)$$

This measure gives an impression of the clustering performance with respect to a user-defined gold standard. However, many biomedical datasets do not provide such a standard. In our case, though, we may utilize the outcome variables (disease annotation and/or the confounding factor annotations, respectively).

### 5.4. Dimension Reduction by Multi-Dimensional Scaling

The visualization of high dimensional data is a challenging and complex task. Carotta integrates the so-called multi-dimensional scaling (MDS), a standard method for this purpose. It aims to find an embedding from the pairwise representation to a space of lower dimension, such that the distances are preserved [36]. Given *N* different objects *z* in a high dimensional space *p*, the objects will be arranged in the low dimensional space *p* in such a way that the pairwise distances are most similar to original distances. Therefore, the objective is to minimize the squared distance of all pairwise distances, Equation (5) [56].

(5) $$S(z_{1},z_{2},\ldots ,z_{N})=\sum_{i\neq j}{\left(\left\|x_{i}-x_{j}\|-\|z_{i}-z_{j}\right\|\right)}^{2}$$

The resulting 2-dimensional or 3-dimensional coordinates can now be visualized by a scatter plot. Another common method for dimension reduction, called principal component analysis, determines the biggest principal components that correspond to the orthogonal direction of larges variance represented by a linear combination of the most varying variables. In contrast, MDS aims to preserve the pairwise distances between each of the two coordinates, influenced by all variables equally. Since these distances are the bases for the clustering, the MDA is a more reasonable choice for this purpose.

### 5.5. Comparison to Existing Software

Several data analysis frameworks have been developed to process, visualize and analyze metabolomics data, particularly for GC/MS data. Some of them focus on pre-processing raw data, but include advanced methods for alignment, peak detection and identification, such as mzMine [57]. Others, like the web application MeltDB, addresses issues concerning metabolomics data storage, sharing, standardization and a binding to R software packages to allow the application of the whole wealth of statistical data analysis tools integrated nowadays in R, which requires programming knowledge, however [58]. More advanced services, such as XCMSOnline [59] and MetaboAnalyst [60], offer advanced statistical analysis techniques. The first, optimized for LC/MS data, offers various parametric and non-parametric test statistics, as well as extended visualizations for meta-analysis (Venn diagrams, for instance). Like Carotta, it offers unsupervised learning techniques and visualization capabilities, mainly principal component analysis (PCA) and HAC. In contrast to Carotta, it does not provide means for systematically exploring adequate measures for internal and external clustering quality, which are essential to evaluate the information content of the clusterings and to pick reasonable clustering parameters/thresholds. The MetaboAnalyst web server also provides access to GC/MS data pre-processing, multivariate statistics and PCA, but focuses mainly on supervised learning and time series analysis afterwards. It is supporting advanced learning methods, such as partial least squares, discriminant analysis or random forest and an evaluation framework, including cross-validation, permutation test and ROC curve analysis, but it neglects features for systematically exploring the results of unsupervised data processing technologies. In contrast, Carotta’s focus lies on the *de novo* detection of confounding factors. It enables the analysis of breath datasets, for instance, to detangle potential biomarkers and confounders in an unsupervised manner.

Existing methods for such multi-dimensional clustering, such as bi-clustering or co-clustering [33,34], do not provide graphical frameworks to systematically explore the parameter space. Carotta, however, allows one to easily design and apply a sequence of various clustering combinations of metabolites and samples and to investigate all results visually and systematically using different validity measures.

We like to emphasize that the main focus of Carotta is breath data analysis, yet its utility is neither limited to MCC-IMS data nor to breath gas profiling. Applications in transcriptomics (gene expression data) or related omics fields are generally possible. Here, we study breath data only, as this kind of data is rich in yet undiscovered confounders emerging from the environment, nutrition or ambient air. Besides systematic confounders breath data might also be prone to various technological sorts of noise. An extensive analysis of their effects is needed, but beyond the scope of this paper.

Unlike all other tools, but MetaboAnalyst, Carotta allows one to directly process a metabolomics peak matrix (independent of the utilized technology). MetaboAnalyst, however, does not support systematic clustering exploration. The MCC/IMS community has established a number of standard procedures for pre-processing, and a set of integrated tools has been developed in the past; see [22,25,61]. As all existing frameworks, Carotta also does not yet support such pre-processing functionality, but offers a flexible plugin architecture, which we will use in the future to implement such features, amongst others.

## Supplementary Files

Supplementary File 1
