# Supplementary material for: Carotta: Revealing Hidden Confounder Markers in Metabolic Breath Profiles
Source: Metabolites. 2015 Jun 10;5(2):344–63. doi: 10.3390/metabo5020344 (PMC4495376; doi:10.3390/metabo5020344)
Supplement: Supplementary File 1 [file metabolites-05-00344-s001.zip › metabolites-80755-supplementary-final/metabolites-80755-supplementary-final.pdf]

Supplementary File

1. Artificial Data Set Generation

The artificial dataset is dedicated to demonstrate and clarify the capabilities of Carotta. It consists of 16 samples and 12 metabolites associated to three metabolite groups. Each of these groups is related to one of the three predefined labels of the samples: (1) "health" (values: healthy (5), disease subtype 1 (5), disease subtype 2 (6)); (2) "smoking" (values: Smoker (9), Non-Smoker(7)); (3) "nutrition" (values: Apple Juice (4), Tee (4), Orange Juice (4), Coffee (4)). The following Tables **??–??** show the corresponding mean and standard deviations used to generate normal distributions from which the artificial data set was sampled.

Table S1. Distribution of metabolite expression mean intensities and their variance (standard deviation) for artificial patients with class label "Health".

Metabolite	Healthy	Subtype 1	Subtype 2	Standard deviation
Metabolite 1	1	4	3	0.25
Metabolite 2	3	6	5	0.1
Metabolite 3	6	9	8	0.5
Metabolite 4	0	3	2	0.7

Table S2. Distribution of metabolite expression mean intensities and their variance (standard deviation) for artificial patients with class label "Nutrition".

Metabolite	Orange Juice	Apple Juice	Tee	Coffee	Standard deviation
Metabolite 1	0	2	4	6	1
Metabolite 2	2	4	6	8	1
Metabolite 3	5	7	9	11	0.5
Metabolite 4	−1	1	3	5	0.75

Table S3. Distribution of metabolite expression mean intensities and their variance (standard deviation) for artificial patients with class label "Smoking".

Metabolite	Non-Smoker	Smoker	Standard deviation
Metabolite 1	−1	5	1
Metabolite 2	1	7	1
Metabolite 3	2	8	2
Metabolite 4	−2	4	0.7

2. Agglomerative Methods for HAC

- **Complete-linkage:** Here the elements with the maximum distance are used:
  $D = \max_{x,y} d(x,y)$
- **Single-linkage:** It is known as "nearest neighbour clustering" because the two elements with the smallest distances are used:
  $D = \min_{x,y} d(x,y)$
- **Ward-linkage:** We define $\bar{X}, \bar{Y}$ as the centre of the clusters X and Y. This distance tries to minimise the variance within the clusters. It tends to build clusters with equal size.
  $D = \frac{d(\bar{X},\bar{Y})}{\frac{1}{|X|}+\frac{1}{|Y|}}$
- **Average-linkage:** Here we use the average distance of all objects.
  $\frac{1}{|X||Y|} \sum_{X,Y} d(x,y)$
- **Centroid-linkage:** This method uses the distance between the centroids of both clusters.

3. F-Measure of the 14 Metabolite Subsets in the COPD Data Set

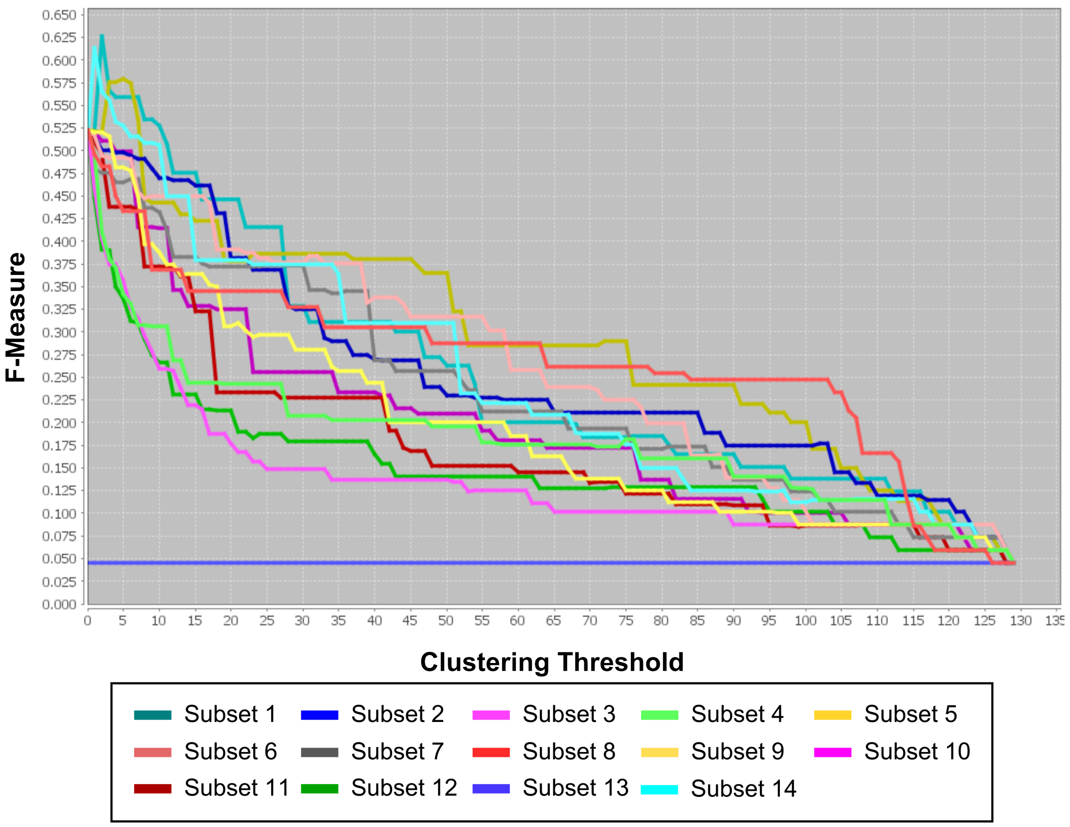

Figure S1. Comparison of the clustering results of the 14 metabolite clusters. The plot shows the F-measure for different clustering thresholds computed against the disease annotation (COPD, COPD with BC, and healthy). Given three groups of patients in the annotation, we are particularly interested in the performance at clustering results at T 3 (x-axis).
