# Supplementary figures and images for: Carotta: Revealing Hidden Confounder Markers in Metabolic Breath Profiles
Source: Metabolites. 2015 Jun 10;5(2):344–63. doi: 10.3390/metabo5020344 (PMC4495376; doi:10.3390/metabo5020344)

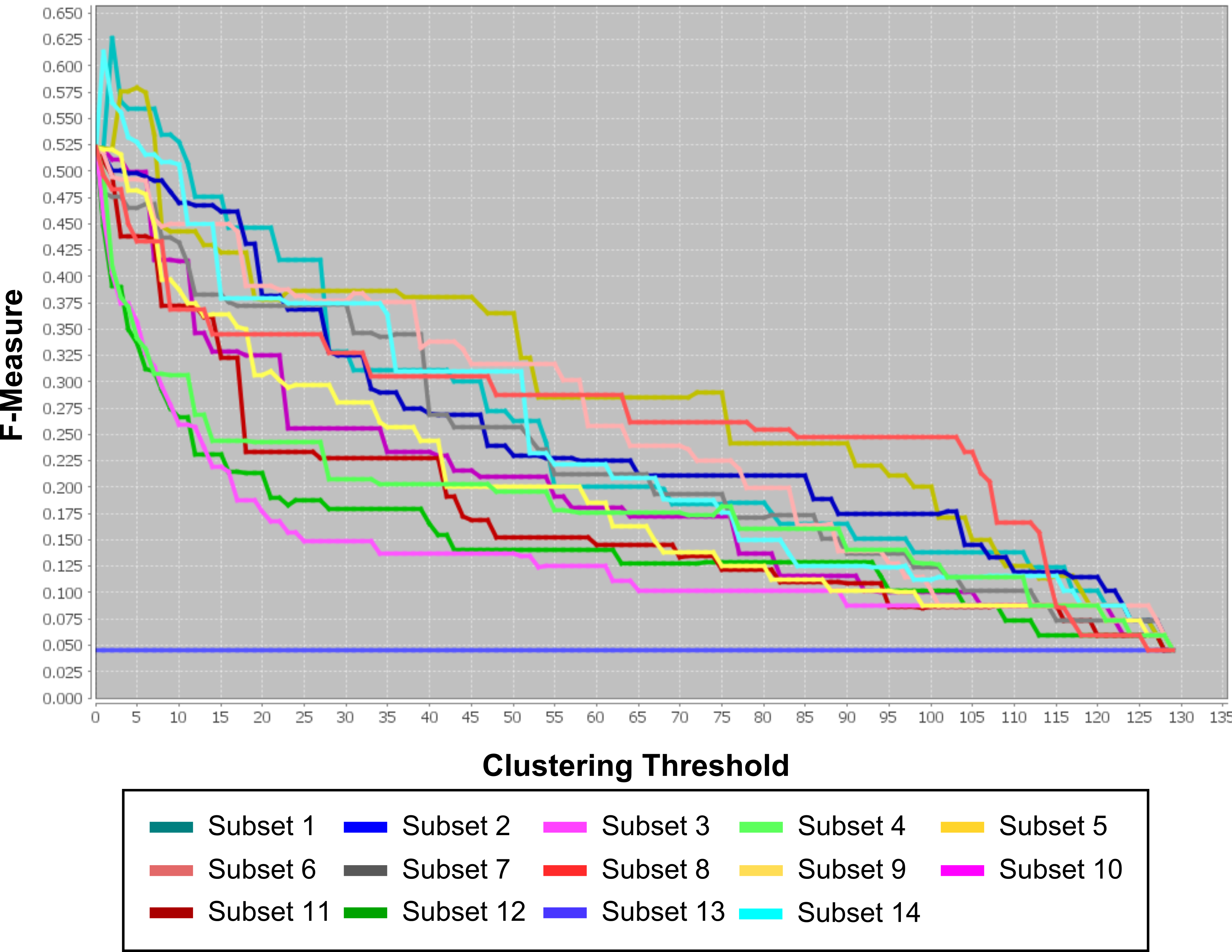

Supplement: Supplementary File 1 [file metabolites-05-00344-s001.zip › metabolites-80755-supplementary-final/figures/fmeasureCOPDALL.png]
